# Exploitation of virgin olive oil by‐products (*Olea europaea* L.): phenolic and volatile compounds transformations phenomena in fresh two‐phase olive pomace (‘alperujo’) under different storage conditions

**DOI:** 10.1002/jsfa.11593

**Published:** 2021-11-04

**Authors:** Lorenzo Cecchi, Marzia Migliorini, Elisa Giambanelli, Valentina Canuti, Maria Bellumori, Nadia Mulinacci, Bruno Zanoni

**Affiliations:** ^1^ Department of NEUROFARBA University of Florence Florence Italy; ^2^ Carapelli Firenze S.p.A. Florence Italy; ^3^ Department of Agricultural Food and Forestry Systems Management (DAGRI), University of Florence Florence Italy

**Keywords:** HS‐SPME‐GC‐MS, secoiridoids, olive mill by‐products, olive pomace storage, nutraceuticals, polyphenols

## Abstract

**BACKGROUND:**

Much effort has recently been spent for re‐using virgin olive oil by‐products as nutraceutical ingredients for human diet thanks to their richness in bioactive phenols, but their management is not easy for producers. We aimed to provide useful information for a better management of fresh olive pomace before drying, by studying the phenolic and volatile compounds transformations phenomena of fresh olive pomace stored under different conditions planned to simulate controlled and uncontrolled temperature conditions in olive oil mills.

**RESULTS:**

The evolution of the phenolic and volatile compounds was studied by high‐performance liquid chromatography‐diode array detector mass spectrometry (HPLC‐DAD‐MS) and headspace solid‐phase microextraction gas chromatography‐mass spectrometry (HS‐SPME‐GC‐MS). The phenolic profile varied rapidly during storage: the verbascoside content decreased about 70% after 17 days even at 4 °C, while the content of simple phenols such as hydroxytyrosol and caffeic acid increased over time. The low temperature was able to slow down these phenomena. A total of 94 volatile organic compounds (VOCs) were detected in the fresh olive pomace, with a prevalence of lipoxygenase (LOX) VOCs (78%), mainly aldehydes (19 490.9 μg kg^−1^) despite the higher number of alcohols. A decrease in LOX volatiles and a quick development of the ones linked to off‐flavors (carboxylic acids, alcohols, acetates) were observed, in particular after 4 days of storage at room temperature. Only storage at 4 °C allowed these phenomena to be slowed down.

**CONCLUSION:**

To preserve the natural phenolic phytocomplex of fresh olive pomace before drying and to avoid off‐flavors development, storage in open containers must be avoided and a short storage in cold rooms (7–10 days) is to be preferred. © 2021 The Authors. *Journal of The Science of Food and Agriculture* published by John Wiley & Sons Ltd on behalf of Society of Chemical Industry.

## INTRODUCTION

About 3.14 million tons of olive oil were produced worldwide during the 2018/2019 crop season, being Spain (39.2%), Italy (10.8%) and Greece (9.6%) the largest manufacturers.^1^ The olive oil extraction process causes the production of several million tons of waste per year.^2^, ^3^ The three‐phase ‘decanter’ centrifuge causes the production of liquid (the olive mill wastewater, or ‘alpechin’) and solid (the three‐phase olive pomace, or ‘orujo’) waste, whereas the more and more used two‐phase ‘decanter’ centrifuge causes the production of a semi‐solid waste, namely the two‐phase olive pomace or ‘alperujo’.^3^, ^4^, ^5^

The olive pomace is recognized as phytotoxic and pollutant for the environment,^5^, ^6^ due to its high amount of phenolic compounds. The latter compounds are transferred in low amounts from olive fruits to olive oil (i.e., < 0.5%)^7^ and the remaining portion is lost in the waste, with the ‘alperujo’ usually richer in phenolic compounds than the ‘orujo’. Several studies also highlighted many health‐beneficial biological properties exerted by phenolic compounds from *Olea europaea* L.^8^, ^9^, ^10^ The European Food Safety Authority approved a health claim for olive oil polyphenols, which attests a beneficial effect if 20 g of olive oil with a phenolic content of at least 5 mg/20 g_oil_ are daily consumed.^11^ Hence, the olive pomace can also be considered as a valuable by‐product for recovering phenolic compounds. Olive pomace has been characterized^3^, ^6^, ^12^, ^13^ and, after drying, successfully proposed as an ingredient for animal diet^14^ and as a nutraceutical ingredient for human consumption.^5^, ^15^, ^16^ For example, despite the bitter character of the olive pomace phenolic compounds, staple foods such as pasta, bread and granola bar fortified with dried olive pomace were largely accepted even by consumers less accustomed to the bitter taste such as the Californian ones.^15^

Several problems hinder a widespread re‐use of ‘alperujo’ as a nutraceutical ingredient for human health. The use of an innovative two‐phase decanter allows overcoming the problem linked to stone residues by separating them from the pulp directly after malaxation, thus reducing hydrolytic and oxidative phenomena.^3^, ^6^ Another problem is given by the high water content, which makes ‘alperujo’ a biologically active matrix, with the consequent risk of chemical and biological degradation phenomena leading to changes/losses in the phenolic profile and to decomposition/hydrolysis of biodegradable organic matter with formation of off‐flavors.^17^, ^18^, ^19^, ^20^, ^21^, ^22^ Therefore, a preservation of olive pomace should be carried out as soon as it is produced. Drying or at least a preliminary freezing of the olive pomace should be used for guaranteeing a suitable olive pomace preservation. Concerning drying, efforts are being spent in search of suitable techniques less expensive than lyophilization (i.e., the drying method of choice up to now) and capable to dry the ‘alperujo’ preserving its nutraceutical properties, mainly at industrial scale.^6^, ^13^, ^23^ Moreover, during olive oil extraction season (i.e., two to three months), the efforts of small and large producers are completely devoted to the olive oil production, and the ‘alperujo’ is usually stored for quite a long period under uncontrolled conditions, allowing degradation phenomena to occur and causing a significant loss of value in view of its re‐use. Therefore, knowledge of the changes occurring during the conservation of the fresh alperujo is necessary to improve the management of this by‐product intended as a new nutraceutical ingredient. Table 1 briefly summarizes the problems hindering the widespread use of alperujo including future lines of action and research.

**Table 1:** Problems hindering a widespread use of olive pomace (alperujo) as ingredient for animal and human consumption

Problem	Details/possible solutions/lines of action	Reference
Presence of stone residual hindering re‐use of alperujo	Use of a particular two‐phase decanter able to separate the pulp from stone and peel residues directly after malaxation. The use of these types of decanter should be considered by all producers that want to re‐use the olive pomace as nutraceutical food ingredient.	Cecchi *et al*., 2018 [6]
Hydrolytic and oxidative phenomena affect fresh olive pomace composition		Lozano‐Sanchez *et al*., 2017 [3]
High moisture content makes fresh olive pomace a perishable matrix	This causes enzymatic degradation of phenols and development of off‐flavors. The problem can be overcome by drying or freezing the fresh olive pomace as soon as possible. The effect of different drying procedures should be investigated.	Sinrod *et al*., 2019 [23]; Brenes *et al*., 2004 [17]; Cecchi *et al*., 2018 [6]; Persuric *et al*., 2020 [13]; Borja *et al*., 2006 [19]
Storage before freezing/drying	This is a very critical aspect: during olive oil extraction season, producers are completely devoted to olive oil, and the ‘alperujo’ is stored for quite long periods under uncontrolled conditions. Studying the best conditions to store the olive pomace before freezing/drying is strongly required	Current manuscript
Drying strategy	Freeze‐drying is certainly the method of choice for a suitable drying of low amounts of this by‐product, but it is a technique quite expensive. Searching for less expensive techniques for drying alperujo at larger scales preserving its nutraceuticals properties is the future lines of research	Cecchi *et al*., 2018 [6]; Sinrod *et al*., 2019 [23]
Acceptability of foods intended for human consumption and fortified with dried olive pomace	When dried alperujo is used to fortify food products for human consumption, the sensory quality of the food is changed (e.g., color, odor, taste), and it is necessary to test the acceptability of the food. Sensory experiment involving consumers from several geographic origins should be carried out in the next future research. When it is instead used in formulation of food supplements, sensory quality evaluation is not requested	Cecchi *et al*., 2019 [15]

The aim of this research was to study the phenolic and volatile compounds transformations phenomena of fresh ‘alperujo’ during storage in the laboratory under different conditions. The different storage conditions were planned to simulate both controlled and uncontrolled temperature conditions in olive oil mills, in order to provide producers with useful information for better management of the ‘alperujo’ before a long‐term preservation step such as drying.

## MATERIAL AND METHODS

### Chemicals

Ultrapure water was from the Milli‐Q‐system (Millipore SA, Molsheim, France). Acetonitrile with HPLC grade was from Panreac (Barcelona, Spain); hexane, formic acid and ethanol were from Sigma‐Aldrich (Steinheim, Germany). Phenolic standards were tyrosol (> 99.5%) and oleocanthal from Sigma‐Aldrich, oleacein from Phytolab (Vestenbergsgreuth, Germany), luteolin‐7‐*O*‐glucoside (> 98%), verbascoside (> 99%), oleuropein (> 98%) and rutin (> 99%) from Extrasynthese (Genay, France). 4‐Methyl‐2‐pentanol (≥ 98.0%) used as internal standard and the 73 external standard for analysis of volatile organic compounds (VOCs) were from Sigma‐Aldrich: their purity was previously reported.^24^ Solutions of VOCs were prepared in refined olive oil free from VOCs.

### Samples

Approximately 80 kg of olive pomace (the ‘alperujo’ sample, *Olea europaea* L.) were collected on November 14, 2019 from the output of a two‐phase ‘decanter’ (Toscana Enologica Mori, Florence, Italy) immediately after processing a batch of olives of the three typical Tuscan cultivars (i.e., Frantoio, Moraiolo, and Leccino) in the province of Florence (Italy). The selected batch was constituted by healthy fruits.

### Experimental plan

Three types of storage of ‘alperujo’ under different laboratory conditions were tested. As soon as a sample arrived in the laboratory, the ‘alperujo’ was homogenized and divided into three aliquots of approximately 25 kg each, which in turn were homogenized and divided into three sub‐aliquots of approximately 5 kg each. The sub‐aliquots were placed in airtight plastic containers (≈15 cm diameter) in a layer of 25 to 30 cm. The containers were stored as follows: (i) one sub‐aliquot in completely filled and closed containers at room temperature, in order to simulate a storage under uncontrolled temperature conditions in absence of air (the closed samples); (ii) one sub‐aliquot in completely filled and closed containers in the fridge at 4 °C, in order to simulate a storage under low and controlled temperature conditions in absence of air (the 4 °C samples); (iii) one sub‐aliquot in open containers at room temperature, in order to simulate a storage under uncontrolled temperature conditions in presence of air (the open samples). The diagram in Scheme S1 of the Supplementary File S1summarizes the experimental plan.

For analysis, aliquots of samples were taken from the bulk of the 4 °C and closed samples, and from the upper layer of the open samples. The volatile compounds were measured at time zero, and after 1, 2, 4, 7 and 10 days of storage. The phenolic compounds were measured at time zero, and after 2, 10 and 17 days of storage.

### Olive pomace water and oil contents

At each sampling date, an aliquot of each ‘alperujo’ sample was freeze‐dried up to constant weight (3 days) to indirectly measure the water content. The freeze‐dried samples were used to measure the oil content, through extraction with hexane.^25^

### Analysis of phenolic compounds

#### Extraction

About 4 g of ‘alperujo’ were extracted twice with 35 mL of ethanol–water, 80:20 *v/v*, homogenizing the mixture for 60 min. After each cycle of extraction, the mixture was centrifuged (0 °C, 10 min, 1667 × *g*) and the supernatant was recovered. The final hydroalcoholic solution was defatted with hexane (50 mL) and evaporated under vacuum. The residue was re‐dissolved in methanol–water, 50:50 *v/v* (8 mL), and the obtained mixture was centrifuged at 13148 × *g*. The supernatant was used for high‐performance liquid chromatography (HPLC) analysis and for acid hydrolysis as follows.

#### Acid hydrolysis of the extract

The phenolic extracts were submitted to acid hydrolysis. Briefly, 200 μL of extract were treated for 2 h at 80 °C in the presence of 400 μL of sulfuric acid (H_2_SO_4_) 1 mol L^–1^. A total of 400 μL of water were then added, solution was centrifuged for 5 min at 13148 × *g* and the supernatant was used for HPLC analysis.

#### HPLC‐DAD‐MS analysis

Phenolic extracts obtained both before and after acid hydrolysis were analyzed with an HP1100 liquid chromatograph provided with autosampler, binary pump and column heater module, and coupled to a diode array detector (DAD; Agilent, Palo Alto, CA, USA). Phenols were separated in a Poroshell 120, EC‐C18 column (150 mm × 3.0 mm, 2.7 μm ps; Agilent) working at 26 °C. The mobile phase (flow 0.4 mL min^−1^) was constituted by acetonitrile (A) and water (B, pH 3.2, formic acid) and elution was performed using the following multistep linear gradient; solvent B varied 95–60% in 40 min, remained at 60% for 5 min, varied 60–0% in 5 min, remained at 0% for 3 min and finally returned to 95% in 2 min. Equilibration time 10 min. Injection volume, 2 μL for extracts before hydrolysis and 20 μL for extracts after hydrolysis. Chromatograms were recorded at 240, 280 and 330 nm. Furthermore, liquid chromatography coupled with mass spectrometry (LC–MS) analysis were performed with HP 1260 MSD provided with a DAD and a mass spectrometry detector (MSD), and with an atmospheric pressure ionization (API)/electrospray interface (Agilent) using the same HPLC method described earlier and the following electrospray ionization (ESI) parameters: nebulizer pressure 1811 Torr; drying gas flow and temperature, 12.0 L min^−1^, 350 °C; capillary voltage 3500 V. Acquisition in full spectrum scan (range 100–1200 Th) in negative ion mode with fragmentor voltage at 200 V.

Quantitative analysis was performed using calibration lines of tyrosol (linearity range 0–1.21 μg; *R* ^2^ = 0.9999) for tyrosol, hydroxytyrosol and their glucosides; oleuropein (0–3.16 μg, *R* ^2^ = 0.9986) for total phenolic content; *p*‐coumaric acid (0–1.57 μg, *R* ^2^ = 0.9987) for comselogoside; luteolin‐7‐*O*‐glucoside (0–1.57 μg, *R* ^2^ = 0.9956) for luteolin; verbascoside (0–1.96 μg, *R* ^2^ = 0.9996) for caffeic acid, β‐OH‐acteoside isomers 1 and 2 and verbascoside. The phenolic compound contents were expressed on a dry matter basis in order to avoid the apparent effects of concentration of phenols due to a potential evaporation of water from the sample surface, mainly in the open samples.

### Analysis of volatile compounds by HS‐SPME‐GC‐MS

The volatile fraction of samples [4.3 g of fresh pomace + 0.1 g 4‐methyl‐2‐pentanol 10.2 mg kg^−1^ in refined olive oil as internal standard (ISTD)] was pre‐concentrated by headspace‐solid phase microextraction (HS‐SPME) and analyzed by gas chromatography‐mass spectrometry (GC‐MS). HS‐SPME and GC‐MS conditions were previously described.^24^ VOCs were tentatively identified comparing peak mass spectrum with those found in the standard NIST08/Wiley98 libraries database (minimum matching factor, 80%) and evaluating their retention index after analyzing a mixture of C9–C30 linear alkanes.^26^ When available,^24^ VOCs identification was confirmed using commercial standards. Quantitation of VOCs was carried out using 4‐methyl‐2‐pentanol as ISTD: this approach was suitable for studying evolution of VOCs over time, as the objective of the study.

### Statistical analysis

For evaluating the effect of type of storage (Storage), time of storage (Time) and their interaction (Storage × Time), two factor analysis of variance (ANOVA) was run for each volatile and phenolic molecule. The significance of the effect of time for each type of storage was also evaluated through one factor ANOVA, followed by application of Fisher's least significant difference (LSD) test to differentiate between mean values. All statistical analyses were performed using Microsoft Excel and the DSAASTAT version 1.1 software.

## RESULTS AND DISCUSSION

At time 0, the oil content of the ‘alperujo’ samples was 11.0 ± 0.7% on dry matter basis and 2.7 ± 0.2% on fresh matter basis; these data were in agreement with recent studies.^23^ The water content of the ‘alperujo’ samples was 75.3% ± 0.8% at time 0 and no significant variation (*P* < 0.05) of the water content occurred in the closed and 4 °C samples during storage; instead, water evaporation caused a significant decrease of water content from the upper layer of the open samples, reaching 65.0% ± 0.9% and 58.1% ± 0.9% after 10 and 17 days of storage, respectively.

Concerning the experimental plan, the effect of light exposure over time was not evaluated in this study: indeed, even in the case of storing conditions in open containers, it can only affect the lipid fraction of the surface of the alperujo sample, which is only a very small part of the sample.

### Characterization of phenolic and volatile compounds profile of the fresh ‘alperujo’ samples

Table 2 shows the contents at time 0 of the main phenolic compounds evaluated by mean of HPLC before acid hydrolysis in the fresh ‘alperujo’ of the Tuscan cultivars, Frantoio, Moraiolo and Leccino. The molecular structures of the evaluated compounds are shown in Supporting Information, Fig. S1, and the chromatographic profile of the fresh ‘alperujo’ is shown in Fig. S2(A).

**Table 2:** Phenolic composition of the alperujo samples at time 0

Compound (mg kg^−1^ dw)	Before hydrolysis	After hydrolysis
Hydroxytyrosol glucoside	764.1 ± 30.6	—
Hydroxytyrosol	2567.2 ± 132.6	8135.6 ± 349.6
Tyrosol glucoside	543.1 ± 24.3	—
Tyrosol	632.0 ± 24.4	3240.0 ± 131.8
Caffeic acid	172.1 ± 13.5	—
β‐OH Acteoside isomer 1	466.2 ± 21.6	—
β‐OH Acteoside isomer 2	625.2 ± 22.9	—
Verbascoside	2165.6 ± 73.2	—
Comselogoside	452.8 ± 18.4	—
Luteolin	433.8 ± 8.1	—
Total phenolic content	94 062.1 ± 5159.0	**—**

Data from three independent determinations are expressed on dry weight (dw) basis.

The typical simple phenols from *Olea europaea* L. (namely tyrosol, hydroxytyrosol and their glycosylated forms) were the most representative compounds, with a prevalence of free hydroxytyrosol (2567.2 mg kg^−1^), followed by tyrosol (632.0 mg kg^−1^) and their glycosylated forms (764.1 and 543.1 mg kg^−1^, respectively), for a total of 4506.4 mg kg^−1^, in agreement with the literature.^23^

The verbascoside‐type group of phenylpropanoids derivatives, containing both hydroxytyrosol and caffeic acid moieties in their molecular structure (Fig. S1), were present in quite an higher total concentration (3257.0 mg kg^−1^) than those reported in previous studies,^3^, ^4^, ^13^, ^23^ with a prevalence of verbascoside (2165.6 mg kg^−1^), followed by the two diastereomers of β‐OH acteoside. The *p*‐coumaroyl‐6′‐secologanoside (comselogoside), bearing the *p*‐coumaric acid moiety in the molecular structure, was present in the amount of 452.8 mg kg^−1^. This compound is usually present in olive fruits but not in olive oil, due to the hydrophilic character given by the glucose moiety^25^; its presence in olive pomace has been already reported in amounts lower than in this study.^3^, ^4^ The flavone luteolin (433.8 mg kg^−1^) and caffeic acid (172.1 mg kg^−1^) were also detected; caffeic acid has been previously found in quite low amounts in two‐phase olive pomace, whereas luteolin was found in variable amounts in the olive pomace.^3^, ^4^, ^13^, ^23^

The total content of tyrosol and hydroxytyrosol (as sum of their free and bound forms) was also measured after acid hydrolysis^6^, ^27^ in order to quantitate the total content of secoiridoid derivatives. These molecules, derived from a series of reactions starting with deglycosylation of oleuropein and ligstroside, cannot be easily quantitated as such due to their poor chromatographic resolution [e.g., dialdehydic form of decarboxymethyl oleuropein aglycone (oleacein), dialdehydic form of decarboxymethyl ligstroside aglycone (oleocanthal), Fig. S2(B,C)]. After hydrolysis, the hydroxytyrosol content was more than double than that of tyrosol (Table 2), meaning that oleuropein derivatives were present in concentration higher than ligstroside derivatives in the ‘alperujo’ samples, in agreement with previous studies.^15^ Due to the variability in the phenolic composition of products from *Olea europaea* L. plants of different cultivars,^28^, ^29^ future research will clarify if this is also true for other cultivars or if for some of them ligstroside derivatives prevail.

Table 3 shows the VOCs content in ‘alperujo’ samples of the selected cultivars at time 0. To the authors’ knowledge, only one previous manuscript reported a characterization of the volatile fraction of olive pomace but, in that case, analysis was performed after samples sterilization at 121 °C.^30^ A total of 94 molecules were detected: 12 carboxylic acids, 11 ketones, one α‐hydroxy‐ketone, 20 aldehydes, five hydrocarbons, 27 alcohols, 15 esters (among which three acetates), one furan and two volatile phenols. Despite the higher number of alcohols, aldehydes were largely the most abundant class of VOCs (19 490.9 μg kg^−1^), followed by alcohols (4680.4 μg kg^−1^) and ketones (1425.9 μg kg^−1^). Esters other than acetates, volatile phenols, furans and α‐hydroxy‐ketones were present in very low amounts. The main contribution to carbonyl compounds amount was given by the lipoxygenase (LOX) pathway‐related VOCs, linked to positive sensory attributes^31^, ^32^: 1‐penten‐3‐one accounted for about 50% of the total ketones content, whereas (*E*)‐2‐hexenal (59.7%), hexanal (28.0%) and (*Z*)‐3‐hexenal (5.0%) accounted for about 92% of the total aldehydes content. The LOX molecules also prevailed among alcohols (approximately 53%), with the main contribution given by (*E*)‐2‐hexen‐1‐ol (17.0%), followed by 1‐penten‐3‐ol (11.4%), (*Z*)‐2‐penten‐1‐ol (8.8%), 1‐hexanol (8.2%), (*Z*)‐3‐hexen‐1‐ol (6.8%) and (*E*)‐2‐penten‐1‐ol (1.0%). The LOX‐VOCs represented 78% of the total VOCs content of the fresh ‘alperujo’, suggesting that the LOX pathway gave the main contribution to the formation of VOCs for at least 2 h after the crushing of the olives.

**Table 3:** Volatile compounds composition of the alperujo samples at time 0

*Aldehydes (μg kg* ^*−1*^ *dw)*	
Acetaldehyde	91.3 ± 19.7
2‐Methylpropanal	66.0 ± 12.4
2‐Methylbutanal	189.6 ± 37.6
3‐Methylbutanal	198.4 ± 36.6
Pentanal	16.8 ± 2.2
Hexanal	5465.2 ± 1498.1
(*E*)‐2‐Pentenal	75.0 ± 14.0
Heptanal	15.5 ± 3.5
(*Z*)‐3‐Hexenal	979.6 ± 221.5
(*E*)‐2 Hexenal	11655.2 ± 2387.6
Octanal	12.4 ± 2.2
(*E*)‐2‐Heptenal	203.7 ± 46.6
Nonanal	79.1 ± 17.5
(*E*,*E*)‐2,4‐Hexadienal	274.6 ± 48.2
(*E*)‐2‐Octenal	7.5 ± 1.8
Decanal	n.d.
(*E*,*E*)‐2,4‐Heptadienal	7.2 ± 1.7
Benzaldehyde	139.5 ± 32.6
(*E*)‐2‐Nonenal	n.d.
(*E*)‐2‐Decenal	8.8 ± 2.4
(*E*,*E*)‐2,4‐Nonadienal	2.5 ± 0.7
(*E,E*)‐2,4‐Decadienal	2.9 ± 1.0
Total aldehydes	19490.9 ± 4387.8
*Ketones (μg kg* ^*−1*^ *dw)*	
Butanone	14.8 ± 2.2
2,3‐Butanedione	85.7 ± 8.9
3‐Pentanone	299.9 ± 33.6
Methyl isobutyl ketone	2.9 ± 0.0
1‐Penten‐3‐one	679.0 ± 129.6
2‐Heptanone	8.6 ± 1.2
2‐Octanone	1.6 ± 0.4
1‐Octen‐3‐one	2.2 ± 0.5
6‐Methyl‐5‐hepten‐2‐one	69.8 ± 23.7
2‐Nonanone	2.5 ± 0.6
4‐Hexen‐2‐one	258.9 ± 30.6
Total ketones	1425.9 ± 230.8
*Alcohols (μg kg* ^*−1*^ *dw)*	
Methanol	1059.5 ± 123.0
Ethanol	709.3 ± 137.6
2‐Butanol	n.d.
1‐Propanol	3.5 ± 1.2
2‐Methyl‐1‐propanol	14.7 ± 2.3
2,2‐Dimethyl‐1‐propanol	3.8 ± 1.4
3‐Pentanol	4.8 ± 0.4
2‐Pentanol	1.0 ± 0.4
1‐Butanol	1.0 ± 0.3
1‐Penten‐3‐ol	536.4 ± 74.1
2‐methyl‐ + 3‐methyl‐1‐butanol	71.9 ± 8.5
1‐Pentanol	49.9 ± 10.3
(*E*)‐2‐Penten‐1‐ol	48.3 ± 5.8
2‐Heptanol	1.5 ± 0.1
(*Z*)‐2‐Penten‐1‐ol	413.2 ± 59.0
1‐Hexanol	383.7 ± 68.5
2‐Methyl‐2,3‐pentanediol	0.6 ± 0.0
(*E*)‐3‐Hexen‐1‐ol	57.1 ± 10.8
(*Z*)‐3‐Hexen‐1‐ol	318.0 ± 46.0
(*E*)‐2‐Hexen‐1‐ol	794.0 ± 122.4
(*E*)‐4‐Hexen‐1‐ol	n.d.
2‐Octanol	n.d.
(*Z*)‐2‐Hexen‐1‐ol	7.4 ± 1.2
1‐Octen‐3‐ol	14.6 ± 3.6
1‐Heptanol	12.9 ± 2.9
4‐Hepten‐1‐ol	0.8 ± 0.3
1‐Octanol	11.6 ± 3.0
2,6‐Dimethyl‐5‐hepten‐1‐ol	n.d.
1‐Nonanol	4.9 ± 1.4
1‐Decanol	n.d.
Benzyl alcohol	51.9 ± 5.8
Phenyl ethanol	104.1 ± 14.1
Total alcohols	4680.4 ± 704.5
*Carboxylic acids (μg kg* ^*−1*^ *dw)*	
Acetic acid	185.5 ± 13.1
Propanoic acid	n.d.
2‐Methyl propanoic acid	2.2 ± 0.3
2,2‐Dimethyl propanoic acid	2.4 ± 0.4
Butanoic acid	2.1 ± 0.4
3‐Methyl butanoic acid	5.2 ± 0.8
2‐Methyl butanoic acid	3.4 ± 0.4
Pentanoic acid	1.4 ± 0.3
(*E*)‐2‐Pentenoic acid	n.d.
Hexanoic acid	88.2 ± 19.9
Heptanoic acid	2.2 ± 0.6
(*E*)‐3‐Hexenoic acid	6.8 ± 1.4
Octanoic acid	7.1 ± 3.2
Nonanoic acid	7.2 ± 3.5
Total carboxylic acids	313.7 ± 44.1
*Acetates (μg kg* ^*−1*^ *dw)*	
Methyl acetate	503.2 ± 116.4
Ethyl acetate	9.6 ± 1.1
Isobutyl acetate	n.d.
2,2‐Dimethyl‐1‐propyl acetate	n.d.
Butyl acetate	n.d.
(*Z*)‐2‐Pentenyl acetate	n.d.
2‐Methylbutyl + 3‐methylbutyl acetate	n.d.
Pentyl acetate	n.d.
Hexyl acetate	0.6 ± 0.1
(*Z*)‐3‐Hexenyl acetate	n.d.
(*E*)‐2‐Hexenyl acetate	n.d.
Heptyl acetate	n.d.
Acetoin acetate	n.d.
Nonyl acetate	n.d.
Total acetates	513.4 ± 117.5
*Other esters (μg kg* ^*−1*^ *dw)*	
Methyl propanoate	1.6 ± 0.3
Ethyl propanoate	n.d.
Ethyl 2‐methylpropanoate	n.d.
Methyl 3‐methylbutanoate	1.1 ± 0.2
Ethyl butanoate	n.d.
Ethyl 3‐methylbutanoate	n.d.
Ethyl pentanoate	n.d.
Methyl hexanoate	28.6 ± 7.5
Ethyl‐3‐methyl‐2‐butenoate	22.5 ± 4.2
Ethyl hexanoate	2.3 ± 0.4
Ethyl tiglate	4.4 ± 0.8
Methyl heptanoate	1.9 ± 0.5
Ethyl (*Z*)‐3‐hexenoate	n.d.
Ethyl heptanoate	1.4 ± 0.3
Methyl octanoate	12.7 ± 7.3
Ethyl octanoate	0.7 ± 0.3
Methyl nonanoate	6.2 ± 1.9
Ethyl nonanoate	n.d.
Methyl decanoate	2.8 ± 1.9
Ethyl decanoate	n.d.
Ethyl benzoate	n.d.
Total esters	86.2 ± 25.6
*Hydrocarbons (μg kg* ^*−1*^ *dw)*	
Heptane	25.0 ± 7.6
Octane	541.3 ± 129.6
Toluene	209.9 ± 61.3
Limonene	3.6 ± 1.0
Styrene	4.7 ± 1.0
Total hydrocarbons	784.5 ± 200.4
*α‐Hydroxy‐ketones (μg kg* ^*−1*^ *dw)*	
1‐Hydroxy‐2‐propanone	n.d.
3‐Hydroxy‐2‐butanone (acetoin)	36.6 ± 5.6
2‐Hydroxy‐3‐pentanone	n.d.
Total α‐hydroxy‐ketones	36.6 ± 5.6
*Volatile phenols (μg kg* ^*−1*^ *dw)*	
Guaiacol	2.5 ± 0.3
Phenol	3.4 ± 0.3
4‐Ethylguaiacol	n.d.
4‐Ethylphenol	n.d.
Total volatile phenols	5.9 ± 0.6
*Furans (μg kg* ^*−1*^ *dw)*	
2‐Pentylfuran	6.3 ± 1.2
Total furans	6.3 ± 1.2

Data from three independent determinations are expressed on dry weight (dw) basis; n.d., not detected.

The amount of other VOCs, including those responsible for off‐flavors, were quite low in the fresh ‘alperujo’ samples, with methanol (1059.5 μg kg^−1^), ethanol (709.3 μg kg^−1^), octane (541.3 μg kg^−1^) and methyl acetate (503.2 μg kg^−1^) as the most abundant ones. Acetic (185.5 μg kg^−1^) and hexanoic (88.2 μg kg^−1^) acids were the most abundant carboxylic acids. Some literature data proposed a relationship between the earlier compounds and some degradation phenomena: microbial spoilage activities were related to methanol formation from pectin compounds, ethanol was related to fermentation of simple sugars by yeasts, followed by an oxidation to acetic acid due to *Acetobacter* species, while octane was related to oleic acid oxidation.^33^, ^34^, ^35^

### Effect of storage conditions on the phenolic profile of ‘alperujo’ samples

**Figure 1:**
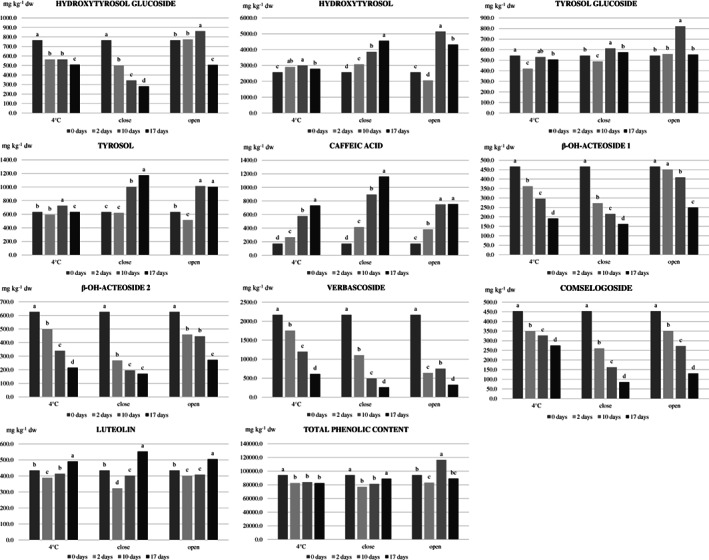
Evolution over time of the content of phenolic compounds in ‘alperujo’ according to type of storage: at 4 °C in the fridge in full and closed containers (4 °C), at room temperature in full closed containers (close) and at room temperature in open containers (open). Data are the mean of three independent measurements and are expressed on dried matter basis. For each molecule and for each type of storage, different letters indicate a different content over time at *P* = 0.05.

Figure 1 shows the variation over time of the phenolic compounds content measured before acid hydrolysis in the ‘alperujo’ samples under different storage conditions. The type of storage, the storage time and their interaction had a significant effect on the variation of phenols (Supporting Information, Table S1). The total phenolic content showed a small and irregular variation under all storage conditions; however, the phenolic profile of the ‘alperujo’ samples changed deeply in a few days, even at 4 °C, determining a quick alteration of the fresh ‘alperujo’ phytocomplex. Chemical and enzymatic hydrolytic phenomena of some phenolic compounds, also favored by the high water content of the ‘alperujo’ samples, could support the earlier behavior.

The verbascoside content strongly decreased over time under all storage conditions, reaching approximately 30% of the initial content after 17 days in the 4 °C samples, and less than 15% of the initial content after 17 days at room temperature in both open and closed samples. Interestingly, after 2 days of storage, the verbascoside content in the 4 °C samples was approximately 80% of the initial one, whereas, in the closed and open samples, it was approximately 50% and 30% of the initial content, respectively. The behavior of the β‐OH‐acteoside diastereomers was quite similar to the verbascoside in the 4 °C and closed samples, whereas the decrease was slower in the open samples: in the latter samples, after 10 days of storage, the content of the sum of the two β‐OH‐acteoside diastereomers was still about 80% of the initial one. The earlier similar behavior of verbascoside and β‐OH‐acteoside diastereomers in the 4 °C and closed samples, which also corresponded to an increase in caffeic acid, suggested a hydrolytic action of esterase on verbascoside and β‐OH‐acteoside diastereomers. Instead, the different behavior observed in the open samples could be due to two concomitant actions: (i) the earlier action of esterase on both β‐OH‐acteoside diastereomers and verbascoside, causing the decrease of the content of all the three compounds; (ii) an enzymatic hydroxylation reaction of verbascoside, causing the decrease of the content of this compound and an increase of the content of the β‐OH‐acteoside diastereomers. This latter action has been previously observed during malaxation of olive pastes at different combination of time and temperature.^36^ These findings suggest that the degradation phenomena occurring immediately after olive crushing are different than those occurring in by‐products after oil extraction, without a crucial role of temperature.

**Figure 2:**
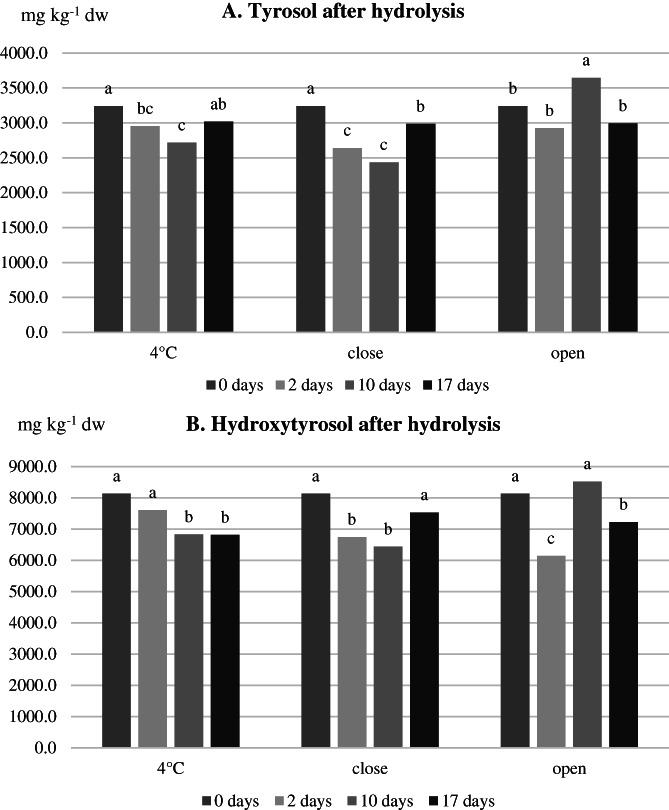
Evolution over time of the content of total tyrosol (A) and hydroxytyrosol (B) after acid hydrolysis in ‘alperujo’ according to the type of storage: at 4 °C in the fridge (4 °C), at room temperature in full and closed containers (close) and at room temperature in open containers (open). Data are the mean of three independent measurements and are expressed on dried matter basis. For each molecule and for each type of storage, different letters indicate a different content over time at *P* = 0.05.

The content of free tyrosol and hydroxytyrosol did not significantly change over time in the 4 °C samples, whereas it constantly increased in the closed and open samples, particularly after 2 days of storage. These increases are probably the result of chemical or enzymatic hydrolytic phenomena on molecules bearing a tyrosol or hydroxytyrosol moiety in their structure, as the deglycosilated forms of oleuropein and ligstroside. This hypothesis was confirmed by the behavior of tyrosol and hydroxytyrosol after hydrolysis (Fig. 2): their content did not show a clear trend over time in none of the storage conditions. No variation of the content of tyrosol glucoside occurred during storage, whereas the content of hydroxytyrosol glucoside decreased over time under all storage conditions.

The comselogoside quickly decreased in the closed samples, reaching approximately 20% of the initial content after 17 days; the decrease was the lowest in the 4 °C samples, reaching approximately 80% of the initial content after 2 days and approximately 60% after 17 days. The decrease in comselogoside content did not correspond to the increase in *p*‐coumaric acid (Fig. S1), similarly to the increase in caffeic acid observed in correspondence of the decrease in verbascoside as reported earlier. The *p*‐coumaric acid was neither detected in fresh ‘alperujo’ samples nor after several days in different conditions, suggesting a different degradation pathway.

### Effect of storage conditions on the volatile profile of ‘alperujo’

A variation of the volatile profile of all ‘alperujo’ samples occurred during storage. Some relevant chromatograms examples of the open samples over time are shown in the Fig. S3.

**Figure 3:**
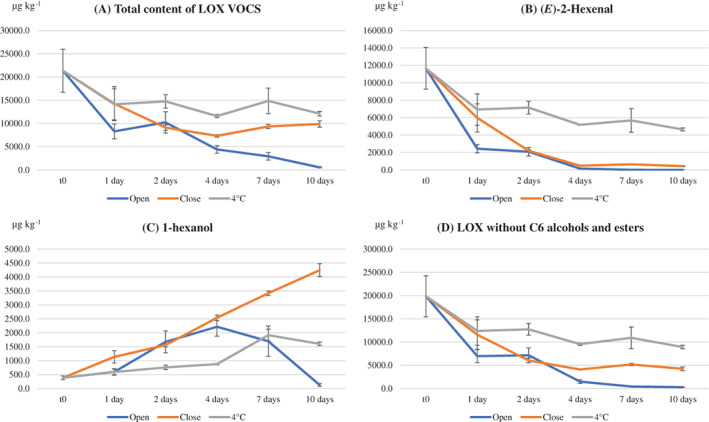
Evolution over time of volatile compounds in ‘alperujo’ according to type of storage: at 4 °C in the fridge (4 °C), at room temperature in full and closed containers (close) and at room temperature in open containers (open). Volatiles originated from the LOX pathway (A), (*E*)‐2‐hexenal (B), 1‐hexanol (C) and volatiles originated from the LOX pathway without C6 alcohols and esters (D). Data are the mean of three independent measurements and are expressed on dried matter basis.

Figure 3(A) shows the variation over time of the total content of LOX‐related VOCs. In the open samples, the total content decreased over time, reaching approximately 2% of the initial content (from ≈ 21 000 to ≈ 500 μg kg^−1^) after 10 days. In the 4 °C samples, the total content significantly decreased only after 1 day, and then it remained in amounts greater than 11 500 μg kg^−1^ for the following period of storage. In the closed samples, the total content of the LOX‐related VOCs decreased up to one‐third of the initial content after 4 days, and then it remained at constant values. As a concern for the single LOX‐related VOCs, the content of all aldehydes, ketones and C5 alcohols decreased over time in all storage conditions; in the open samples, the earlier compounds content reached a value close to zero after 10 days, as in the case of the (*E*)‐2‐hexenal content (Fig. 3(B)). The C6 alcohols contents (e.g., 1‐hexanol, (*E*)‐2‐hexen‐1‐ol, (*Z*)‐3‐hexen‐1‐ol), showed a different trend, exemplified in Fig. 3(C) for the 1‐hexanol, which increased for the first 4 days in all storage conditions, with the highest rate in the open and closed samples. Then, it started to decrease in the open samples likely due to the volatility of these molecules, whereas its increase continued in the closed and 4 °C samples. The earlier‐mentioned behavior may be related to activity of the alcohol dehydrogenase (ADH), an enzyme involved in the LOX pathway that reduces aldehydes to alcohols,^31^ whose activity causes the transformation of the C6 aldehydes, abundant in the fresh sample, in C6 alcohols. Interestingly, high concentrations of C6 alcohols have sometimes been associated with negative sensory attributes in virgin olive oils.^37^, ^38^ The LOX esters, not detectable in the fresh ‘alperujo’ samples, only appeared in low amounts in the last days of storage in the open and closed samples. Figure 3(D) shows the content variation of the LOX VOCs without considering the earlier C6 alcohols and esters, that is the behavior of those LOX VOCs that can be considered as an index of the VOCs linked to positive odor quality was considered. A fast decrease of the earlier compounds content already occurred in the first 2 days under all storage conditions; only the storage conditions at 4 °C allowed slowing down of the decrease of the earlier compounds.

**Figure 4:**
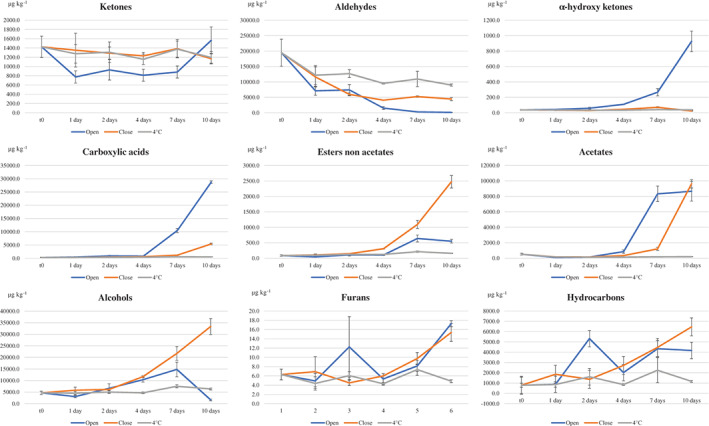
Evolution over time of the sum of VOCs belonging to different chemical classes in ‘alperujo’ according to type of storage: at 4 °C in the fridge (4 °C), at room temperature in full and closed containers (close) and at room temperature in open containers (open). Data are the mean of three independent measurements and are expressed on dried matter basis.

Figure 4 shows the variation over time of the VOCs in terms of total content of specific chemical classes. Volatile phenols are not shown in Fig. 4, because their amount remained very low over the entire storage period; only 4‐ethyl phenol, not detected at time 0, started to slightly increase after 7 days in the open samples.^17^ The furans and ketones seemed not suitable to evaluate the effect of storage conditions, since the furans content was very low over storage and the ketones content only varied in the open samples without a clear trend (Fig. 4). The behavior of aldehydes was similar to the trend of (*E*)‐2‐hexenal (Fig. 3(B)); an exception occurred at the end of storage in the closed samples, mainly due to hexanal, which was still approximately 3000 μg kg^−1^ after 10 days, and to the slight increase of benzaldehyde and acetaldehyde.

A clear effect of the storage conditions was pointed out for the other VOCs (Table S2); this effect can be related with a microbial spoilage of ‘alperujo’. The carboxylic acids quickly increased in the open samples starting after the fourth day of storage and reached very high amounts (Fig. 4): 21 310.7 μg kg^−1^ of acetic acid, 3112.8 μg kg^−1^ of 3‐methyl butanoic acid, 1800.6 μg kg^−1^ of hexanoic acid, 980.6 μg kg^−1^ of 2‐methyl propanoic acid and 967.6 μg kg^−1^ of 2‐methyl butanoic acid. In the closed samples, the increases became significant after 7 days; acetic acid was again the compound that reached the highest amount (3818.7 μg kg^−1^), followed by hexanoic acid (1138.1 μg kg^−1^). The prevalence of acetic acid in aerobic conditions could be due to the action of bacteria as *Acetobacter* and some yeasts, which oxidize ethanol mainly in aerobic conditions^39^; a similar process may be hypothesized for the branched acids, which increase in open samples coincides with the decrease of the corresponding alcohols. The strong increase in the content of carboxylic acids was in agreement with previous papers reporting increases in acidity and volatile acids when ‘alperujo’ was stored in open air ponds.^19^, ^40^

The total content of alcohols reached very high amounts during storage (Fig. 4). Their increase started after 2 days of storage and was similar in the closed and open samples up to the fourth day of storage; then, the earlier increase became much more pronounced in the closed samples, whereas in the open ones the content of alcohols quickly decreased after 7 days. In particular, ethanol reached the highest amount (18 897.2 μg kg^−1^), followed by 1‐hexanol (4247.4 μg kg^−1^), 2‐methyl + 3‐methyl‐1‐butanol (3382.8 μg kg^−1^), phenyl ethanol (1745.9 μg kg^−1^) and 2‐methyl propanol (798.4 μg kg^−1^). The increase of ethanol in both aerobic and anaerobic conditions could be due to yeasts and hetero‐fermentative bacteria through alcoholic fermentation. 2‐Methyl + 3‐methyl‐1‐butanol, 2‐methyl propanol and phenyl ethanol were probably produced by yeasts^30^, ^41^ through deamination or transesterification of the amino acids isoleucine, leucine, valine and phenylalanine, respectively.^33^, ^39^

The increase of acetates was faster in the open than in the closed samples, whereas the contrary was observed for other esters (Fig. 4). Acetates reached quite high amounts (approximately 8000 μg kg^−1^), with a clear prevalence of methyl and ethyl acetate; their increase in the closed samples appeared 3 days late than in the open ones. The sum of other esters reached contents up to 2500 μg kg^−1^ in the closed samples, with a prevalence of ethyl hexanoate (1022.2 μg kg^−1^), ethyl octanoate (439.4 μg kg^−1^) and methyl hexanoate (319.6 μg kg^−1^). Increasing contents of these esters were already detected during microbial fermentation of olive pomace^30^ and table olives.^41^, ^42^

Other VOCs that significantly increased due to detrimental biological phenomena were an α‐hydroxy ketone (acetoin), and two hydrocarbons: styrene and octane. The increase of acetoin almost coincided with the increase of α‐hydroxy ketones and was only observed in the open samples, likely due to the action of bacteria (Fig. 4). Octane showed a slight increase in the 4 °C samples, and a faster increase in the closed and open samples, with a delay of 5 days in the latter case (Fig. S4). Styrene did not increase in the 4 °C samples, while started quickly increasing in both open and closed samples after approximately 2 days (Fig. S4), likely due to L‐phenylalanine deamination and decarboxylation of cinnamic acid by yeasts.^30^, ^42^, ^43^

## CONCLUSIONS

In order to prevent degradation phenomena of ‘alperujo’ during the storage before the drying process, the suitability of some storage conditions similar to those applicable in the mill, was studied in this research. This step is crucial to evaluate the potential feasibility of olive pomace as a nutraceutical ingredient for human diet.

Hydrolytic phenomena caused fast variations of the phenolic profile of ‘alperujo’ samples during different storage conditions. The storage at low temperature was able to slow down these phenomena in the first 2 days of storage, limiting the decrease in the main phenolic compounds, which, at room temperature, was at least 50% for the most abundant phenolic compounds. Volatility and microbial spoilage influenced the VOCs profile of ‘alperujo’ samples during storage: a decrease of the LOX‐related VOCs and an increase of off‐flavors occurred. The low temperature (i.e., 4 °C) was able to avoid both decreases of positive odor attributes and increases of off‐flavors for at least 10 days of storage. On the contrary, at room temperature an increase of the VOCs linked to microbial spoilage and off‐flavors was already observed after 2 days of storage; the earlier increase became very strong after 4 days during a storage in the presence of air and after 7 days during storage in the absence of air.

Overall, the results of this study, obtained using ‘alperujo’ samples of three typical Tuscan cultivars (i.e., Frantoio, Moraiolo, and Leccino), allowed proposing some recommendations to olive oil producers to preserve ‘alperujo’ products. The first choice is to use a cold room for storing ‘alperujo’ for a few days (i.e., 7–10 days) before drying. An alternative is to use a cold room to keep ‘alperujo’ in closed and filled containers for the shortest possible time (i.e., 1–2 days) before drying. Finally, the use of open containers should be avoided.

## CONFLICT OF INTEREST

The authors declare no conflict of interest.

## Supporting information


**Appendix S1**. Process diagram of the experimental plan.
**Figure S1.** Chemical structure of the main phenolic compounds detected in alperujo samples.
**Figure S2.** Chromatographic profile at 280 nm of (A) the fresh alperujo sample at time 0 and after 10 days in open and 4 °C conditions, (B) the commercial standard of the dialdehydic form of decarboxymethyl oleuropein aglycone (oleacein), (C) the commercial standard of the dialdehydic form of decarboxymethyl ligstroside aglycone (oleocanthal).
**Figure S3.** Total ion current (TIC) chromatograms of the volatile profile of the olive pomace at time 0 (A) and after storage in open containers after 2 days (B) and after 10 days (C). All the three chromatograms are reported at the same scale.
**Figure S4.** Evolution over storage of octane and styrene according to type of storage: at 4 °C in the fridge (4 °C), at room temperature in full close containers (Close) and at room temperature in open containers (Open). Data are the mean of three independent measurements and are expressed on dried weight basis.
**Table S1**. The *P*‐values evaluated by two factor analysis of variance (ANOVA) for each phenolic molecule. The two factors were the type of storage (Storage) and storage time (Time), and the two‐way interaction Storage × Time was also considered. Significant values at *P* < 0.05 are in italic
**Table S2.** Volatile organic compounds (VOCs) data processing of alperujo samples. The *P*‐values calculated for each VOC by two factor analysis of variance (ANOVA), where the two factors were the storage time (Time) and the type of storage (Storage). The two‐way interaction Time × Storage is also reported. Non‐significant values (*P* > 0.05) are in italic.
