# Supplementary material for: Exploitation of virgin olive oil by‐products (Olea europaea L.): phenolic and volatile compounds transformations phenomena in fresh two‐phase olive pomace (‘alperujo’) under different storage conditions
Source: J Sci Food Agric. 2021 Nov 4;102(6):2515–25. doi: 10.1002/jsfa.11593 (PMC9298029; doi:10.1002/jsfa.11593)
Supplement: Supplementary file 1 — Appendix S1. Process diagram of the experimental plan. Figure S1. Chemical structure of the main phenolic compounds detected in alperujo samples. Figure S2. Chromatographic profile at 280 nm of (A) the fresh alperujo sample at time 0 and after 10 days in open and 4 °C conditions, (B) the commercial standard of the dialdehydic form of decarboxymethyl oleuropein aglycone (oleacein), (C) the commercial standard of the dialdehydic form of decarboxymethyl ligstroside aglycone (oleocanthal). Figure S3. Total ion current (TIC) chromatograms of the volatile profile of the olive pomace at time 0 (A) and after storage in open containers after 2 days (B) and after 10 days (C). All the three chromatograms are reported at the same scale. Figure S4. Evolution over storage of octane and styrene according to type of storage: at 4 °C in the fridge (4 °C), at room temperature in full close containers (Close) and at room temperature in open containers (Open). Data are the mean of three independent measurements and are expressed on dried weight basis. Table S1. The P‐values evaluated by two factor analysis of variance (ANOVA) for each phenolic molecule. The two factors were the type of storage (Storage) and storage time (Time), and the two‐way interaction Storage × Time was also considered. Significant values at P < 0.05 are in italic Table S2. Volatile organic compounds (VOCs) data processing of alperujo samples. The P‐values calculated for each VOC by two factor analysis of variance (ANOVA), where the two factors were the storage time (Time) and the type of storage (Storage). The two‐way interaction Time × Storage is also reported. Non‐significant values (P > 0.05) are in italic. [file JSFA-102-2515-s001.pdf]

Supplementary file of the manuscript

Exploitation of virgin olive oil by-products (*Olea europaea* L.): phenolic and volatile compounds transformations phenomena in fresh two-phase olive pomace ("alperujo") under different storage conditions

Lorenzo Cecchi[a], Marzia Migliorini[b], Elisa Giambanelli[b], Valentina Canuti[c], Maria Bellumori[a], Nadia Mulinacci[a], Bruno Zanoni[c]

[a] Department of NEUROFARBA, University of Florence, Via Ugo Schiff 6, 50019 Sesto F.no, Florence, Italy

[b] Carapelli Firenze S.p.A., Via Leonardo da Vinci 31, Tavarnelle Val di Pesa, 50028, Firenze, Italy.

[c] Department of Agricultural, Food and Forestry Systems Management (DAGRI), University of Florence, Piazzale Delle Cascine 16, 50144, Florence, Italy.

ORCID

Lorenzo Cecchi: 0000-0002-9332-704X

Nadia Mulinacci: 0000-0001-7873-5259

Elisa Giambanelli: 0000-0002-4164-7770

Bruno Zanoni: 0000-0002-9020-6883

*Corresponding author: Nadia Mulinacci

Tel.:+.39 055 4573773 E-mail: nadia.mulinacci@unifi.it

Scheme S1. Process diagram of the experimental plan

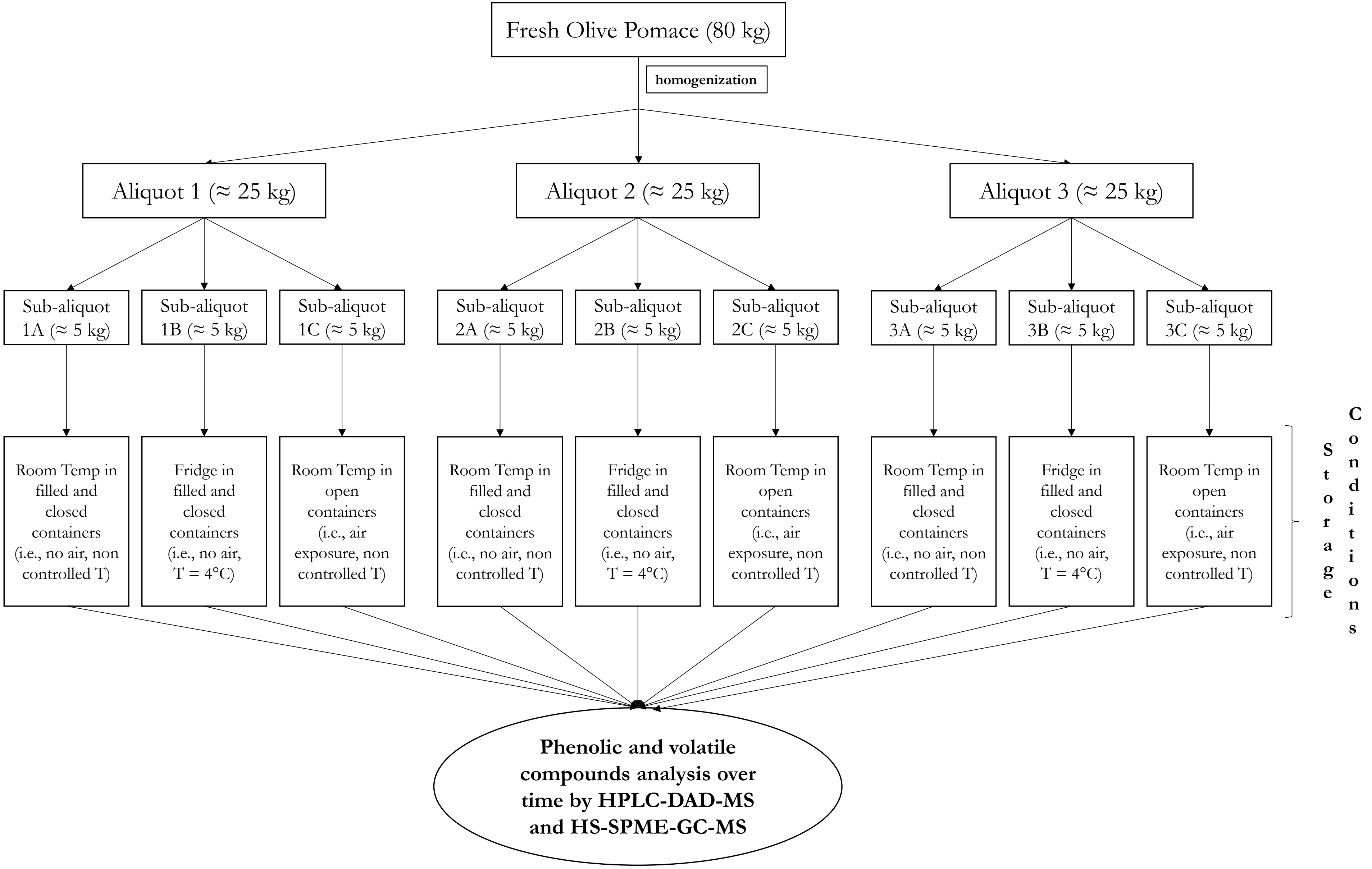

Figure S1. Chemical structure of the main phenolic compounds detected in alperujo samples.

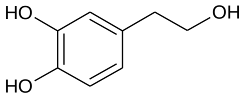

Hydroxytyrosol

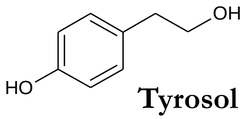

Tyrosol

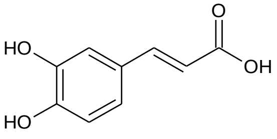

Caffeic acid

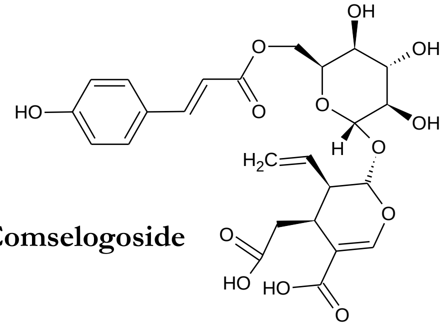

Comselogoside

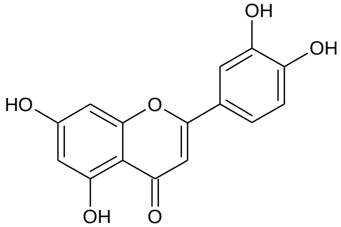

Luteolin

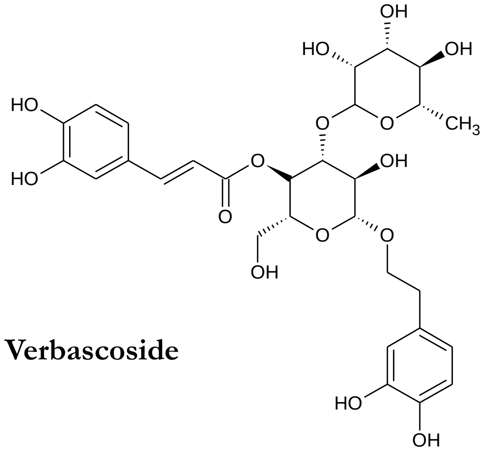

Verbascoside

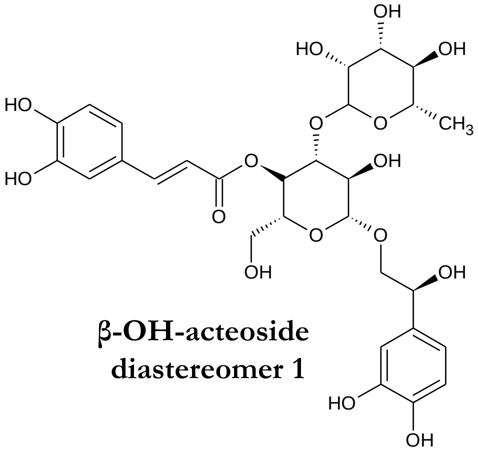

β-OH-acteoside diastereomer 1

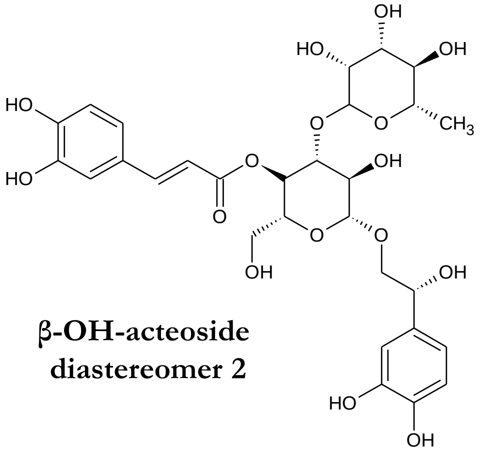

β-OH-acteoside diastereomer 2

Figure S2. Chromatographic profile at 280 nm of A) the fresh alperujo sample at time 0 and after 10 days in open and 4°C conditions, B) the commercial standard of the dialdehydic form of decarboxymethyl oleuropein aglycone (oleacein), C) the commercial standard of the dialdehydic form of decarboxymethyl ligstroside aglycone (oleocanthal).

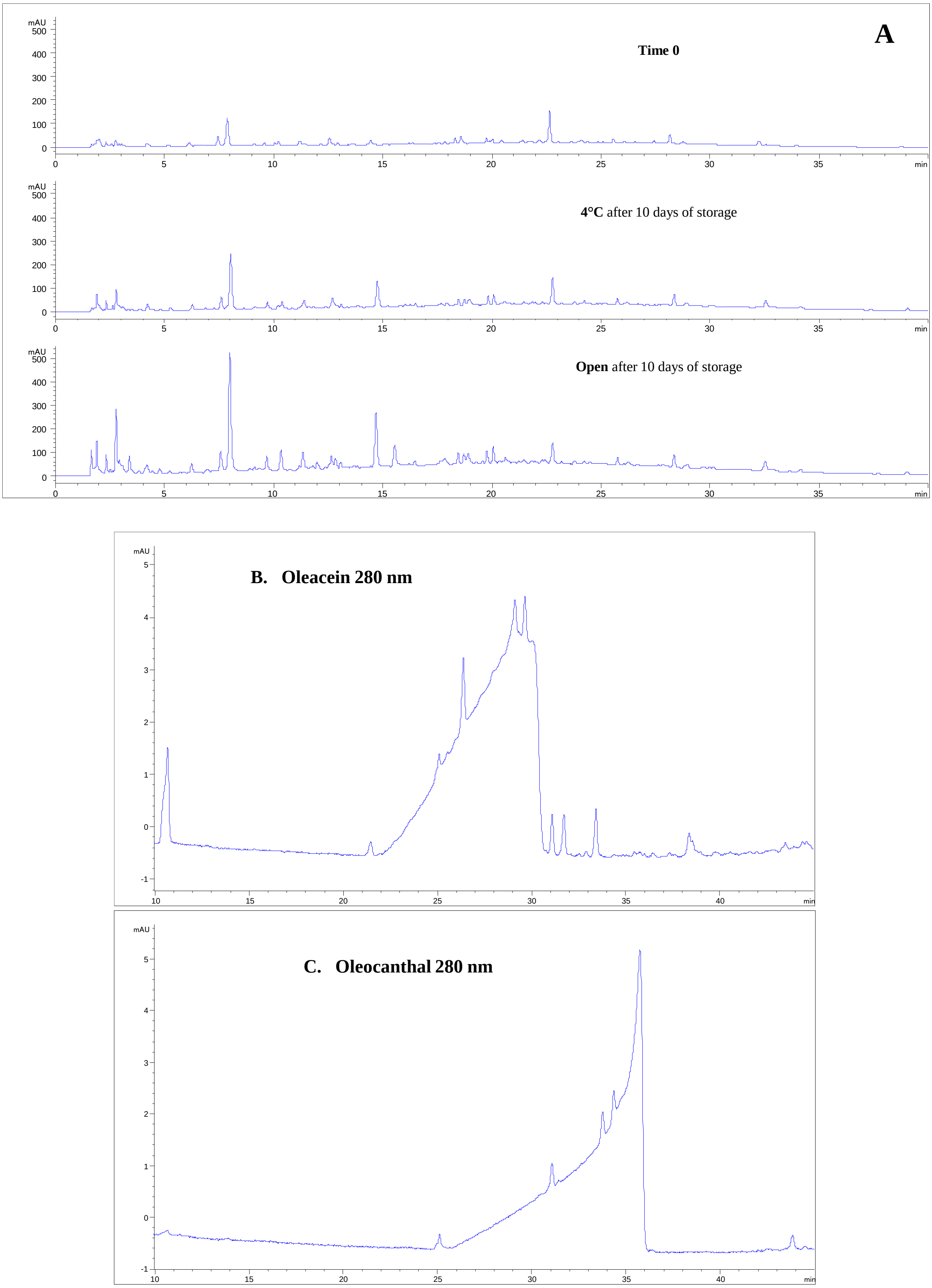

Figure S3. Total Ion Current (TIC) chromatograms of the volatile profile of the olive pomace at time 0 (A) and after storage in open containers after 2 days (B) and after 10 days (C). All the three chromatograms are reported at the same scale.

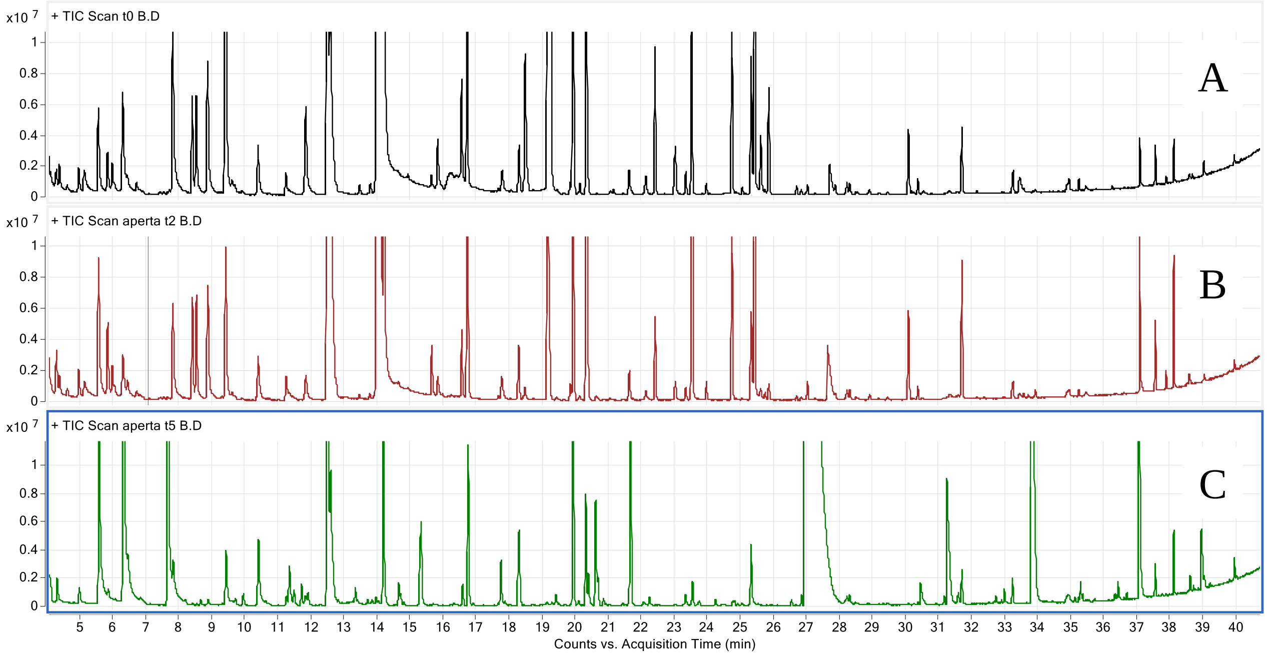

Figure S4. Evolution over storage of octane and styrene according to type of storage: at 4°C in the fridge (4°C), at room temperature in full close containers (close) and at room temperature in open containers. Data are the mean of 3 independent measurements and are expressed on dried weight basis.

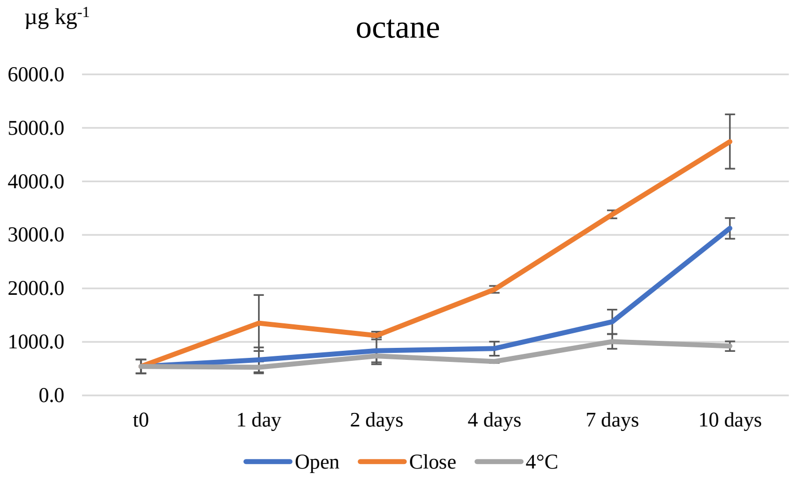

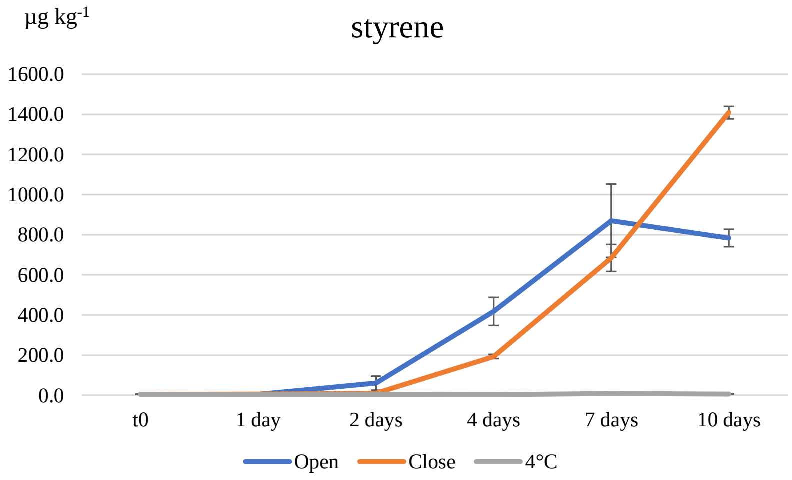

Table S1. *p*-values evaluated by two factor ANOVA for each phenolic molecule. The two factors were the type of storage (Storage) and storage time (Time), and the two-way interaction Storage × Time was also considered. Significant values at $p < 0.05$ are in italic

	Storage	Time	Storage x Time
hydroxytyrosol glucoside	*8.4E-13*	*1.9E-13*	*7.4E-09*
hydroxytyrosol	*1.2E-11*	*8.9E-18*	*2.3E-14*
hydroxytyrosol after hydrolysis	*1.3E-06*	0.18	*1.9E-06*
tyrosol glucoside	*5.5E-09*	*3.4E-10*	*3.1E-07*
tyrosol after hydrolysis	*2.1E-04*	*4.3E-05*	*9.0E-06*
tyrosol	*3.2E-13*	*1.3E-18*	*1.8E-12*
caffeic acid	*4.4E-20*	*4.3E-31*	*1.6E-16*
β-OH-acteoside 1	*2.7E-11*	*1.4E-17*	*2.8E-06*
β-OH-acteoside 2	*1.9E-07*	*2.8E-15*	*2.2E-04*
verbascoside	*7.6E-14*	*1.1E-24*	*7.0E-11*
comselogoside	*1.5E-12*	*2.8E-20*	*2.7E-08*
luteolin	0.43	*3.7E-14*	*3.4E-05*
TPC	*5.1E-05*	*2.3E-05*	*1.3E-05*

Table S2. VOCs data processing of alperujo samples. *p*-values calculated for each VOC by two factor ANOVA, where the two factors were the storage time (**time**) and the type of storage (**storage**). The two-way interaction time×storage is also reported. Non-significant values ($p > 0.05$) are in italic.

	Storage	Time	Storage x Time
**Acetic acid**	2.87333E-38	3.72917E-41	5.62416E-41
**3-Methyl butanoic acid**	2.52404E-34	2.86583E-38	1.80117E-40
**(*E*)-2-Pentenoic acid**	2.60434E-31	2.0849E-34	4.59378E-37
**Pentanoic acid**	2.66132E-30	2.73578E-33	1.66388E-34
**Propanoic acid**	1.22328E-28	1.73116E-30	1.56937E-32
**Methyl 3-methylbutanoate**	2.03372E-25	1.79038E-30	8.82867E-33
**2-Methyl propanoic acid**	4.98891E-28	2.96907E-30	1.56048E-32
**Acetoin acetate**	2.18257E-24	2.00839E-28	1.07438E-31
**2-Methyl butanoic acid**	1.06836E-23	4.96913E-28	1.50919E-29
**3-Pentanol**	1.87933E-22	2.25974E-27	1.1635E-29
**1-Octen-3-ol**	5.82112E-24	3.10772E-27	1.87237E-29
**Methyl acetate**	1.86333E-21	5.24335E-27	7.71643E-27
**(*E*)-3-Hexenoic acid**	1.2207E-21	1.98607E-25	2.57614E-26
**2-methyl-1-butanol+3-methyl-1-butanol**	2.9211E-22	8.98558E-25	2.64265E-24
**4-Hepten-1-ol**	4.23567E-28	6.30893E-24	1.40723E-26
**Ethyl benzoate**	1.07885E-21	2.35976E-23	2.34736E-25
**1-Octen-3-one**	1.79422E-20	2.93569E-23	4.40336E-24
**Ethyl hexanoate**	9.62267E-21	1.45063E-22	1.04466E-24
**Ethyl-(*Z*)-3-hexenoate**	4.64807E-16	1.23234E-21	9.5398E-26
**Methyl hexanoate**	5.17013E-11	2.0117E-21	3.27154E-10
**Hexanoic acid**	1.20397E-11	2.57463E-21	4.54472E-14
**Phenyl ethanol**	3.20778E-16	6.12805E-21	1.86376E-19
**Styrene**	1.30511E-15	8.61611E-21	7.84042E-16
**Nonyl acetate**	7.66464E-15	1.02628E-20	7.78033E-19
**Butanoic acid**	9.11509E-16	2.68329E-20	9.44188E-17
**1-Heptanol**	1.32354E-24	7.20754E-20	4.85246E-21
**Octanoic acid**	3.72728E-18	1.02791E-19	5.54223E-18
**1-Propanol**	2.44563E-20	2.51465E-19	1.6293E-21
**Hexyl acetate**	2.32887E-13	3.02165E-19	2.26572E-20
**Ethyl acetate**	2.28162E-12	5.19429E-19	1.11605E-19
**2-Methyl-1-propanol**	2.83673E-15	5.79771E-19	3.26686E-20
**Heptanoic acid**	1.5779E-14	9.353E-19	7.80402E-20
**Ethyl 2-methylpropanoate**	8.75989E-14	4.52388E-18	7.21527E-15
**Pentyl acetate**	4.84896E-11	1.68031E-17	1.56413E-18
**1-Nonanol**	7.3287E-17	1.77986E-17	5.22497E-16
**1-Decanol**	1.5631E-19	2.02826E-17	4.94009E-18
**2-Methyl-2,3-pentanediol**	2.05751E-18	9.31283E-17	3.35937E-18
**(*Z*)-2-Pentenyl acetate**	3.62277E-12	9.41489E-17	1.19475E-16
**Ethyl 3-methylbutanoate**	1.00484E-10	3.74199E-16	1.1266E-11
**2-Methylbutyl acetate + 3-methylbytyl acetate**	6.06872E-08	1.81113E-15	6.31398E-13
**2-Hydroxy-3-pentanone**	9.36231E-16	3.78008E-15	1.64627E-16
**Octane**	5.81433E-13	4.40872E-15	1.69403E-08
**(*E*,*E*)-2,4-Hexadienal**	8.31398E-06	6.29808E-15	0.51512448
**Ethyl nonanoate**	1.20573E-13	8.52192E-15	2.05218E-14
**Ethyl octanoate**	8.81802E-15	1.09819E-14	2.09877E-16
**2,2-Dimethyl-1-propanol**	7.41256E-17	3.13559E-14	4.89792E-13

**(*Z*)-3-Hexenyl acetate**	1.67812E-08	3.6027E-14	1.64593E-15
**Limonene**	0.047314201	8.70377E-14	0.005347179
**Ethyl decanoate**	3.76224E-13	8.99621E-14	1.09778E-15
**Benzyl Alcohol**	1.69957E-10	9.61069E-14	3.42946E-13
**1-Hexanol**	3.07861E-12	1.12244E-13	7.45523E-11
**Methyl propanoate**	2.94579E-10	1.64078E-13	4.01576E-10
**1-Butanol**	3.11612E-08	1.81544E-13	5.55057E-10
**2,2-Dimethyl propanoic acid**	3.71133E-10	2.4585E-13	6.49333E-14
**3-Hydroxy-2-butanone (acetoin)**	2.15772E-13	7.49771E-13	8.6821E-16
**Heptyl acetate**	4.97871E-06	8.75673E-13	3.0088E-08
**Methyl octanoate**	1.80574E-17	1.11211E-12	5.74551E-15
**(*Z*)-3-hexenal**	0.000160964	3.2284E-12	*0.652685924*
**Ethyl butanoate**	1.02561E-08	5.86089E-12	7.89313E-13
**(*E*)-2 Hexenal**	1.36725E-06	6.48132E-12	0.27562446
**2-Octanone**	8.39845E-07	7.8548E-12	7.98519E-05
**Ethyl 3-methyl-2-butenoate**	1.52636E-05	1.14437E-11	*0.251118742*
**Isobutylacetate**	1.65955E-06	1.229E-11	1.51702E-11
**Ethyl propanoate**	1.12307E-07	1.27068E-11	3.09163E-12
**1-Penten-3-one**	0.031401343	2.68036E-11	*0.710576889*
**1-Pentanol**	5.82448E-15	3.38804E-11	3.49085E-12
**Ethyl heptanoate**	7.42789E-08	3.80106E-11	3.71183E-10
**Ethanol**	3.44778E-11	4.50196E-11	2.45636E-12
**1-Hydroxy-2-propanone**	3.55261E-09	8.21599E-10	2.12795E-12
**(*E*)-2-Pentenal**	1.31277E-05	2.25531E-09	*0.251458378*
**2,2-Dimethyl-1-propyl acetate**	1.33887E-09	2.43726E-09	1.20159E-11
**Methyl decanoate**	4.6237E-12	3.43057E-09	5.05552E-11
**(*E*)-2-Hexen-1-ol**	2.57136E-12	3.98599E-09	4.32823E-11
**(*E*)-2-Penten-1-ol**	1.17579E-08	6.03044E-09	8.91969E-05
**1-Octanol**	5.66564E-12	7.76138E-09	7.1712E-10
**Methyl heptanoate**	0.000532332	1.11892E-08	6.97416E-06
**(*E*)-3-Hexen-1-ol**	7.88218E-17	2.08154E-08	4.00032E-10
**2-Pentanol**	*0.33304922*	3.5473E-08	0.000926408
**4-Ethyl-phenol**	1.8675E-07	3.66565E-08	2.77354E-10
**2,3-Butanedione**	8.17689E-06	4.0649E-08	0.000337648
**Nonanoic acid**	1.99196E-08	1.9425E-07	1.85567E-09
**Ethyl pentanoate**	0.000783374	6.01108E-07	1.04444E-08
**(*Z*)-2-Hexen-1-ol**	2.62659E-15	6.40125E-07	5.98001E-08
**(*Z*)-3-Hexen-1-ol**	4.0564E-08	2.41197E-06	4.41281E-09
**(*Z*)-2-Penten-1-ol**	8.3516E-07	4.15549E-06	0.022741744
**2-Heptanol**	0.017573029	6.51461E-06	0.002214252
**Hexanal**	0.004939952	2.67279E-05	*0.104184313*
**2-Heptanone**	*0.840768764*	0.000124289	*0.085356588*
**Octanal**	0.001382303	0.000175684	*0.213637054*
**3-Methylbutanal**	0.002716883	0.000194814	0.010794553
**Heptanal**	0.004086507	0.000195405	0.037380242
**(*E*)-2-Heptenal**	0.000107487	0.000203516	*0.087413602*
**(*E*)-2-Octenal**	0.00078261	0.000711534	*0.141736992*
**(*E*,*E*)-2,4-Nonadienal**	0.000930442	0.001208263	0.032599937
**2-Methylbutanal**	0.002970931	0.001446163	0.023243709
**2-Methylpropanal**	0.013816763	0.001957445	*0.080941771*
**Methyl isobutyl ketone**	0.001025987	0.002037944	0.000178994
**Pentanal**	*0.128395638*	0.002474495	0.007157624
**1-Penten-3-ol**	5.12522E-05	0.002780428	*0.420032735*
**(*E*,*E*)-2,4-Heptadienal**	0.000254386	0.003066809	*0.142757234*

**(*E*)-2-Decenal**	0.010327312	0.003249809	*0.073592893*
**Methanol**	9.30286E-09	0.00674298	0.025769557
**4-Hexen-2-one**	2.64845E-08	0.014508132	0.007102205
**3-Pentanone**	0.00460309	0.019540282	4.3788E-05
**Methyl nonanoate**	4.01902E-05	0.022199507	0.008174644
**Acetaldehyde**	0.006019301	0.022964923	2.57121E-08
**(*E*,*E*)-2,4-Decadienal**	0.003967724	0.037448943	*0.566879943*
**Nonanal**	0.046950542	*0.052879679*	*0.362236777*
**6-Methyl-5-hepten-2-one**	*0.06526592*	*0.074896564*	*0.656242764*
**Benzaldehyde**	9.60959E-08	*0.079269703*	9.05797E-08
**Heptane**	*0.129495071*	*0.20436331*	*0.679727785*
**Butanone**	0.003522836	*0.255692685*	0.006072708
**Toluene**	*0.241279217*	*0.290491842*	*0.535406712*
